# Elevated thyroid stimulating hormone is associated with elevated cortisol in healthy young men and women

**DOI:** 10.1186/1756-6614-5-13

**Published:** 2012-10-30

**Authors:** Kimberly N Walter, Elizabeth J Corwin, Jan Ulbrecht, Laurence M Demers, Jeanette M Bennett, Courtney A Whetzel, Laura Cousino Klein

**Affiliations:** 1 Department of Biobehavioral Health, 219 Biobehavioral Health Bldg, The Pennsylvania State University, University Park, PA, 16802, USA; 2 Nell Hodgson Woodruff School of Nursing, Emory University, Atlanta, GA, 30322, USA; 3 Department of Pathology, H158 Pathology, The M.S. Hershey Medical Center, Hershey, PA, 17033, USA

**Keywords:** TSH, Free T3, Free T4, Cortisol, Subclinical hypothyroidism

## Abstract

**Background:**

Recent attention has been given to subclinical hypothyroidism, defined as an elevation of TSH (4.5-10 uIU/L) with T4 and T3 levels still within the normal range. Controversy exists about the proper lower limit of TSH that defines patients in the subclinical hypothyroidism range and about if/when subclinical hypothyroidism should be treated. Additional data are needed to examine the relationship between markers of thyroid function in the subclinical hypothyroidism range, biomarkers of health and ultimately health outcomes.

**Objective:**

We aimed to assess the relationship between serum TSH levels in the 0.5-10 uIU/L range and serum cortisol in a cohort of healthy young men and women without clinical evidence of hypothyroidism. Based on data in frank hypothyroidism, we hypothesized that serum TSH levels would be positively correlated with serum cortisol levels, suggesting derangement of the cortisol axis even in subclinical hypothyroidism.

**Methods:**

We conducted a cross sectional study in 54 healthy, young (mean 20.98 +/− 0.37 yrs) men (19) and women (35). Lab sessions took place at 1300 hrs where blood was drawn via indwelling catheter for later assessment of basal serum TSH, free T3, free T4, and cortisol levels.

**Results:**

All but 1 participant had free T3 levels within the normal reference intervals; free T4 levels for all participants were within the normal reference intervals. Linear regression modeling revealed that TSH levels in the 0.5-10 uIU/L were significantly and positively correlated with cortisol levels. This positive TSH-cortisol relationship was maintained below the accepted 4.5 uIU/L subclinical hypothyroid cutoff. Separate regression analyses conducted by systematically dropping the TSH cutoff by 0.50 uIU/L revealed that the TSH-cortisol relationship was maintained for TSH levels (uIU/L) ≤4.0, ≤3.5, ≤3.0, and ≤2.5 but not ≤2.0. Linear regression modeling did not reveal a relationship between free T3 or free T4 levels and cortisol levels.

**Conclusions:**

Results suggest a positive relationship between TSH and cortisol in apparently healthy young individuals. In as much as this relationship may herald a pathologic disorder, these preliminary results suggest that TSH levels > 2.0 uIU/L may be abnormal. Future research should address this hypothesis further, for instance through an intervention study.

## Background

Thyroxine (T4) and triiodothyronine (T3), together referred to as thyroid hormones, play an important role in basal metabolism and the functioning of almost all tissues and systems in the body [1]. In addition to T4 and T3, thyroid stimulating hormone (TSH) secretion typically is maintained within relatively narrow limits via a sensitive negative feedback loop in which TSH stimulates the synthesis and release of thyroid hormones, that in turn negatively feed back to the hypothalamus and anterior pituitary to limit further TSH release. Reduced thyroid hormone levels existing together with elevated TSH are an indication that the response of the thyroid gland to TSH is impaired, i.e. primary hypothyroidism.

Recent attention has been given to subclinical hypothyroidism, defined as a TSH elevation with T4 and T3 levels still within the normal range. Subclinical hypothyroidism is a common disorder; two large population-based studies revealed that 4% to 8.5% of individuals without known thyroid disease actually have subclinical hypothyroidism as evidenced by a mildly elevated TSH (i.e., 4.5-10 uIU/L) [2,3]. Complicating matters is the current controversy about the proper lower limit of TSH that defines patients in the subclinical hypothyroidism range (in other words, the upper limit of the normal reference range for TSH) [4-8]. In apparently healthy populations, the TSH distribution is skewed towards the lower end of the reference range, with the mean value typically being around 1.5 uIU/L, but with the range extending from 0.5–4.5 uIU/L [8]. Therefore it is possible that seemingly healthy individuals with TSH levels in the upper end of this range may in fact have elevated TSH in response to early thyroid gland failure and also should be considered to have subclinical hypothyroidism. This possibility is further supported by the fact that many patients with TSH levels in the 3.0–4.5 uIU/L range are positive for antithyroid antibodies [6,8].

Untreated hypothyroidism can lead to increased body weight, cognitive dysfunction, fatigue, abnormal serum lipids, coronary heart disease, and for women recurrent miscarriage, infertility, and possibly delayed cognitive development in their children [1,9,10]. Much controversy still exists regarding the extent to which clinically meaningful consequences result from untreated subclinical hypothyroidism or are due to TSH values in the upper range of “normal” [4-8]. Indeed, by definition the only abnormality found with subclinical hypothyroidism is a mild to modest TSH elevation that is more sensitive than either free T3 or free T4 in reflecting this condition [4]. Most clinical guidelines recommend either close observation or a therapeutic trial of levothyroxine in patients with TSH values in the 4.5-10 uIU/L range and simply observation when the TSH result is in the range of 2.5-4.5 uIU/L [5,10]. These recommendations are based on lack of conclusive evidence in either range of definitive disease. Intervention studies that examine neurocognitive symptoms in patients identified with suggestive symptoms at baseline, as well as intervention studies that focus on long-term outcomes such as atherosclerosis, have not been carried out.

More research is therefore clearly needed in this area. This preliminary study assessed the relationship between serum TSH levels in the 2.5-10 uIU/L range and serum cortisol in a cohort of healthy young men and women with no clinical history of thyroid disease or other underlying health conditions. It has long been known that frank hypothyroidism causes elevated cortisol levels, presumably due to both decreased clearance and blunted negative feedback of cortisol on the hypothalamic-pituitary-adrenal axis [11]. In the present study we hypothesized that serum TSH levels would be positively associated with serum cortisol levels even in the subclinical hypothyroidism range.

## Materials and methods

### Study participants

Participants were recruited through advertisements in the local newspaper and flyers posted in the local community. Research staff through telephone-screening interviews determined eligibility. Exclusion criteria included a history of smoking (<1 year ago); diagnosed heart disease, diabetes, stroke or other neurological disorders or depression; significant medication use (blood pressure, beta-blockers, inhaled beta agonists, hormonal contraceptives, corticosteroid use within prior three months, psychotropic medication use within prior eight weeks; psychiatric hospitalization within past year; and body mass index (BMI; kg/m^2^) > 32. BMI was confirmed at the beginning of the study session. Women with a partial or complete hysterectomy, tubal ligations, history of menstrual irregularities, and who were pregnant or lactating within the past 12 months were also excluded from the study.

Thirty-five healthy women and 19 men, 18–30 years of age (mean age 20.98 +/− 0.37 years), were eligible. Women were randomly assigned to participate during the late luteal (N=18) or follicular (N=17) phase of their menstrual cycle because of menstrual phase effects on cortisol levels [12]. Sex steroid assays confirmed cycle phase with mean estradiol and progesterone levels for the late luteal phase at 123.94 ± 22.72 pg/mL and 18.12 ± 3.41 ng/mL, respectively, and follicular phase levels at 49.31 ± 6.13 pg/mL and 4.40 ± 0.82 ng/mL, respectively. Thirty-eight (70.4%) of the participants identified themselves as Caucasian, 4 (7.4%) African American, 4 (7.4%) Asian, 2 (3.7%) Hispanic, and 6 (10.7%) self-described as “other.” The Pennsylvania State University’s Institutional Review Board reviewed and approved all study procedures.

### Procedure

Lab sessions took place at 1300 hrs at The Pennsylvania State University General Clinical Research Center. Following informed consent and the measurements of body weight and height, a nurse practitioner confirmed health status and study eligibility. An indwelling catheter was then inserted and, and after a 10-minute acclimation period a 20cc blood sample was drawn and allowed to sit at room temperature for 15 minutes. Samples were centrifuged at 1500 X g at 4 degrees C for 15 minutes. Serum was stored at −80 C until hormone analysis was performed. Participants were compensated for their time.

#### Hormonal analyses

Cortisol, TSH, estradiol, and progesterone levels were determined at The Pennsylvania State University’s GCRC using commercially available enzyme immunoassay kits (Diagnostic Systems Laboratories, Inc., Webster, Texas). Low-end assay sensitivity for cortisol, TSH, estradiol and progesterone were as follows: 0.1 ug/dL (cortisol), 0.01 uIU/L (TSH), 7 pg/mL (estradiol), and 0.13 ng/mL (progesterone). Intra-assay and inter-assay imprecision for all analytes averaged less than 10%; all samples were tested in duplicate in a single assay batch. Duplicate test values that varied by more than 5% were subject to repeat testing. The average of the duplicate test results is reported below. Serum free T3 (FT3) and free T4 (FT4) levels were determined by radioimmunoassay with reagents obtained from Siemens Medical Solutions (Hollidaysburg, PA).

#### Statistical analyses

Statistical analyses were performed using SPSS, version 17.0 (Chicago, IL, US). Means are reported plus or minus standard error of the mean. Statistical significance levels were set at α=0.05 and two-tailed tests were used.

## Results

Serum free T4 levels for all participants were within the normal reference interval (i.e., 0.7-1.8 ng/dl see Table 1). Serum free T3 levels fell within the normal reference interval (i.e., 2.0-5.0 pg/mL) for all but 1 participant whose level was elevated (11.5 pg/mL). We included this participant in our analyses because FT3 results are less reliable than FT4 results due to the low concentration found in the circulation and TSH and FT4 were within the normal range for this participant (TSH=3.61 uIU/L; FT4=1.6 ng/dl). It is important to note that the following results do not change as a result of removing this participant from the analyses. Overall, these FT3 and FT4 data confirm the absence of overt clinical hypothyroidism in our participants.

**Table 1:** **Mean serum TSH (uIU/L), free T3 (pg/mL), free T4 (ng/dL), and cortisol (ug/dL), and BMI (kg/m**^**2**^**) in men (N=19) and in women during the luteal (N=18) and follicular (N=14) phases of the menstrual cycle (± standard error of the mean)**

	**Men (N=19)**	**Women luteal (N=18)**	**Women follicular (N=14)**
TSH (uIU/L)	3.25 ± 0.44	2.34 ± 0.32	2.21 ± 0.26
Free T3 (pg/mL)	3.04 ± 0.11	2.89 ± 0.11	3.29 ± 0.52
Free T4 (ng/dL)	1.28 ± 0.04	1.25 ± 0.05	1.28 ± 0.04
Cortisol (ug/dL)	14.77 ± 1.21	9.35 ± 0.87	10.94 ± 1.17
BMI (kg/m^2^)	23.37 ± 0.72	23.73 ± 0.80	23.23 ± 0.82

Cortisol and TSH results were not normally distributed (i.e., were skewed). Therefore, a natural logarithmic transformation was applied to the cortisol and TSH values e.g., [13,14], which resulted in a normal distribution of the data. Thus, the transformed data were used for analyses, but the raw data are reported in Table 1 for clarity. Two serum TSH level ranges were tested in the analyses: [1] levels below the cutoff for overt hypothyroidism (<10 uIU/L) and [2] levels below the cutoff for subclinical hypothyroidism (<4.5 uIU/L). Overall, serum TSH levels varied widely among participants who otherwise presented to the study as healthy. Consistent with U.S. prevalence rates [2,3], approximately 9% of the sample population (N=5) demonstrated mildly elevated TSH levels (4.5-10 uIU/L); these participants typically would be categorized as having subclinical hypothyroid disease in a medical setting [10]. Three additional participants (all women in the follicular phase) had serum TSH levels indicative of overt clinical hypothyroidism (TSH ≥10 uIU/L) and were excluded from analyses because we were interested in the relationship between TSH and cortisol among healthy individuals. It is important to note that the following results interpretation did not change as a result of this exclusion. Serum TSH levels from the final cohort (N=51) ranged from 0.86-9.57 uIU/L (mean 2.64 ± 0.22 uIU/L). Table 1 lists the average TSH and cortisol results by hormone status group (± standard error of the mean) for the final group. Cortisol levels were in the normal range for early afternoon.

Separate one-way analyses of variance (ANOVAs) confirmed that TSH, FT3, FT4 levels, as well as body mass index (BMI), across the 3 hormone status groups were similar (see Table 1); data were therefore collapsed across hormone status groups for the subsequent analyses. Body mass index was included as a covariate in the analyses but it did not affect the statistical significance of the models. Thus, BMI was not included in the final models.

Simple linear regression modeling revealed that serum TSH levels ≤ 10 uIU/L significantly predicted serum cortisol levels [β=0.33, *t*(50)=2.80, *p*<0.05*.*. This TSH-cortisol relationship also held for subjects who were nominally euthyroid (i.e., below the subclinical hypothyroid cutoff, i.e., TSH ≤ 4.5 uIU/L) [β=0.49, *t*(46)=3.35, *p*<0.05]. To further explore the TSH-cortisol relationship, we ran separate regression analyses by systematically dropping the TSH cutoff by 0.50 uIU/L. The positive TSH-cortisol relationship was maintained for TSH levels: [1] ≤ 4.0 uIU/L [β=0.53, *t*(45)=3.51, *p*<0.05], [2] ≤ 3.5 uIU/L [β=0.23, *t*(40)=2.38, *p*<0.05], [3] ≤ 3.0 uIU/L [β=0.34, *t*(37)=3.19, *p*<0.05], and [4] ≤ 2.5 uIU/L [β=0.68, *t*(30)=2.89, *p*<0.05]. Serum TSH levels below 2.0 uIU/L were not significantly associated with serum cortisol levels [β=0.33, *t*(18)= 6.53, *ns*. In other words, serum cortisol levels among individuals with TSH levels >2.0 uIU/L (N=31) were significantly higher than among those individuals with TSH levels ≤ 2.0 uIU/L (N=20) [13.83 ± 0.93 ug/dL vs. 8.66 ± 0.59 ug/dL, respectively, F(1,48)=14.24, p<0.0001]. There was no relationship observed between either FT3 or FT4 and cortisol levels.

## Discussion

We examined the relationship between TSH levels and cortisol in a preliminary study of young, healthy adults without known thyroid disease or other underlying health conditions. The positive relationship between serum TSH and cortisol levels in a healthy population is a compelling new finding that is consistent with and extends the observation that frankly hypothyroid patients have frankly elevated cortisol levels [11].

These preliminary results raise important questions – such as whether this relationship is pathologic or phy siologic and what the mechanism(s) involved in this relationship may be. While in frank hypothyroidism, it is hypothyroidism that causes elevation of cortisol by reducing peripheral disposal and blunting feedback of cortisol on the hypothalamic-pituitary-adrenal axis [11], our cross sectional data do not elucidate whether the same mechanisms hold true for TSH levels in the high normal and low elevated range. Thus more definitive population-based and intervention studies are now needed to confirm this finding and answer these questions.

Another potential explanation for the positive TSH-cortisol relationship is that hypothyroidism - subclinical or clinical - is associated with subtle metabolic stress. Metabolic stress could be imposing an effect on the adrenocorticotropin hormone-adrenal axis leading to an increase in stress hormone (i.e., cortisol) release and production. This hypothesis should be confirmed through the measurement of other stress hormones including the catecholamines, norepinephrine/epinephrine, and/or prolactin.

Although limited in sample size, our findings demonstrate that a positive relationship exists between TSH and cortisol that is maintained down to a TSH level of 2.5 uIU/L (but not below). This observation raises the possibility that negative health effects of mild, subclinical hypothyroidism with mild to modest elevations in TSH may begin at levels much lower than those currently considered abnormal based on assigned normal reference range values with an upper reference level of 4.5 uIU/L.

Chronic elevations in serum cortisol and hypothyroidism (including subclinical hypothyroidism) have been separately linked with increased rates of depression, anxiety, and poor cognitive functioning e.g., [15-17]. Thus, the association between TSH levels and cortisol suggests at least the possibility of a novel pathway through which hypothyroidism (both clinical and subclinical) may promote poor mental health; or hypothyroidism and an elevated cortisol level could be synergistic on mental health.

It is possible that the relationship described in this paper is physiologic rather than representative of pathology. Indeed, a failure to observe a relationship between TSH and cortisol for TSH < 2.5uIU/L may be a matter of methodology (assay accuracy at the lower levels, statistical power) rather than an indication that a relationship does not exist at those lower levels. Additional, larger clinical studies are needed that also include repeat TSH, FT3, FT4, cortisol, and catecholamine measurements to validate the reliability of the TSH-cortisol relationship.

A strength of this preliminary study is that we also measured FT3 and FT4 levels in this cohort of young, healthy individuals. Interestingly, while our results demonstrate that subtle elevations in TSH are associated with higher basal cortisol levels, this relationship is not apparent for FT3 or FT4 levels. One plausible explanation for the lack of an association between FT3 or FT4 and cortisol is that changes in FT3 and FT4 are slow to reflect subclinical hypothyroidism in the circulation because of adjustments made at the end organ level in both synthesis and metabolism of thyroid hormones [4]. In addition, changes in TSH are 10 times more sensitive in reflecting an abnormality in thyroid hormone homeostasis compared to FT3 or FT4 [4]. Similar to our finding, other studies of subclinical hypothyroidism typically report a relationship between the biomarker of interest (e.g., cholesterol) and TSH, but not FT3 or FT4 [18]. However, it is still possible that mechanistically the relationship that we are here reporting is indeed between T3 and/or T4 and cortisol, but that measurement of FT4 and FT3 in blood is not sensitive enough to represent effects at the tissue level.

Our results and those of Iranmanesh et al. [11] for frank hypothyroidism suggest effects of the thyroid axis on the adrenal axis where hypothyroidism causes hypercortisolemia. It is of interest to consider these observations together with the well-established opposite effects, i.e. effects of the adrenal axis on thyroid function. Thus when cortisol levels are manipulated through pathologic as well as physiologic ranges, a negative relationship is found between cortisol and TSH. Both exogenous and endogenous (i.e. Cushing’s Syndrome, stress) corticosteroids suppress TSH [19-22] while low cortisol levels elevate TSH [23,24]. These studies all taken together suggest a physiologic feedback loop where lower thyroid function increases cortisol, but cortisol feeds back to reduce TSH; this hypothesis is consistent with the observations that in the case of primary hypothyroidism (elevated TSH) cortisol is elevated, but in the setting of primarily elevated cortisol TSH is suppressed. Clearly further clinical studies, for instance studies that incrementally administer TSH and evaluate cortisol levels, are needed in order to better understand the mechanisms involved in the TSH-cortisol relationship.

In summary these initial data, that add to what is already known about frank hypothyroidism and cortisol, demonstrate a potentially important relationship between TSH and cortisol in apparently healthy young individuals. The finding that this relationship appears to hold in the controversial TSH range of 2.5-10 uIU/L, but not below, is compelling and requires further scientific and clinical investigation.

## Abbreviations

TSH: Thyroid stimulating hormone; T4: Thyroxine; T3: Triiodothyronine; FT4: Free T4; FT3: Free T3; BMI: Body mass index.

## Competing interests

The authors have no competing interests to declare.

## Authors’ contributions

KNW developed the hypotheses and data analytic strategy, analyzed the data and drafted the manuscript. LCK and EJC designed the experiment and obtained funding. JU and LMD provided crucial clinical and scientific endocrine and thyroid functioning expertise. JU and LCK assisted with data analytic strategies. LMD provided funding and conducted FT4 and FT3 assays. JMB and CAW assisted with running the experiment, as well as TSH and cortisol assays. All authors made intellectual revisions to the entire manuscript, edited all sections of the manuscript and approved this version of the submitted manuscript.
